# Social network sites (SNS) an archetype of techno-social stress: A systematic review

**DOI:** 10.1016/j.heliyon.2024.e41119

**Published:** 2024-12-10

**Authors:** January F. Naga, Ryan A. Ebardo

**Affiliations:** a Department of Information Technology, MSU-Iligan Institute of Technology, Iligan City, Philippines; b Department of Information Technology, De La Salle University, Philippines

**Keywords:** Coping, SNS stressor, SNS strain, Systematic review, Technostress

## Abstract

**Background:**

Social Networking Sites (SNS) are widely used platforms known for both their hedonic and social connectivity benefits. Although there is considerable interest in understanding how personal technostress affects individual well-being, a significant gap remains in the systematic exploration of this topic within the literature.

**Methods:**

This review systematically examined 41 empirical studies from Scopus and PubMed published between 2014 and 2023, following PRISMA guidelines, and assessed for methodological quality using the Mixed Method Appraisal Tool. Only English-language articles were considered to avoid translation complexities. Studies were categorized and coded using the Stressor-Strain-Outcome (SSO) model to examine demographic variations in stressor impacts and coping mechanisms.

**Results:**

The findings reveal a complex interplay of technological, behavioral-technological, and social stressors. Technological complexities often exacerbate social stressors like FoMO (Fear of Missing Out) and social overload, resulting in psychological (e.g., anxiety, depression), behavioral (e.g., reduced engagement), and physical strains (e.g., sleep disruptions). Demographic factors significantly influence stress responses, with younger users and women particularly affected. Key intervention strategies identified include digital detox practices, mindfulness techniques, and user-centered SNS design modifications.

**Conclusion:**

This review emphasizes the need for targeted approaches to mitigate SNS-induced technostress to foster balanced, health-oriented digital engagement. Future research should focus on developing comprehensive frameworks to address technostress impacts on diverse user groups better and support sustainable digital well-being practices.

## 1 Introduction

The integration of Information Technology (IT) into various facets of daily life has broadened its utilization for personal purposes, dramatically increasing the personal consumption of social networking sites (SNS), news services, and digital content dramatically [1,2]. This widespread adoption, however, is not without its drawbacks. One significant adverse effect is technostress—a type of stress experienced from the use of IT [3] —which arises not just in workplace settings but also in personal and leisure activities.

Technostress in personal IT use often involves a complex interplay of stressors and strains that vary from those experienced in organizational settings [4,5]. Personal use of technology, typically voluntary and devoid of organizational support, emphasizes hedonic experiences over utilitarian benefits, leading to potentially unmitigated exposure to technostress [2].

SNS platforms such as Facebook, Instagram, Twitter (now known as X), and apps like WhatsApp and Snapchat facilitate social interaction and networking. However, unique challenges are also introduced. Users face a constant barrage of updates and the pressure of social comparisons, contributing to technostress [6,7]. This stress is further amplified by the personal, often emotional nature of the content shared, which can range from minor daily updates to significant life events [8].

Recent studies have begun to address technostress in personal contexts, examining how social interaction complexities on SNS can intensify stress, particularly when combined with technological demands. For instance, Lin, Fu and Zhou [9] found that social overload—stemming from constant interactions, notifications, and feedback loops—compounds the effects of technological complexities, such as frequent updates and privacy concerns. This interaction creates a cycle where social and technological stressors amplify each other, resulting in increased anxiety and reduced mental well-being [3]. These findings point to the importance of examining technostress outside traditional workplace settings, considering the diverse range of stressors that SNS users encounter in non-work environments.

Recognizing the personal context of IT use as a dominant feature in modern life; it becomes essential to examine the specific sources and impacts of technostress within these settings [10]. While extensive studies exist on the occupational origins and consequences of technostress, research on its manifestation in personal and voluntary IT use remains limited [11]. Previous reviews primarily emphasize workplace or academic environments, neglecting the unique stressors and strains of non-work-related SNS use, where users lack institutional support and engage for hedonic purposes. For instance, studies have identified stressors like social overload, self-comparison, and complex SNS interactions that are distinct in personal contexts, yet these are underexplored in terms of their cumulative impact on user well-being [12,13]. Furthermore, while there is evidence of SNS use affecting mental health, comprehensive research is needed to understand how technostress mechanisms specifically contribute to these effects across diverse demographics [14,15].

This review seeks to provide a comprehensive analysis of SNS-induced technostress, emphasizing the varied stressors, strains, and coping strategies across different demographic groups. By examining technological and social stressors, this review addresses a critical gap in understanding how SNS technostress manifests in non-work settings, where users engage voluntarily and lack institutional support. Furthermore, it explores how individuals cope with these unique stressors, identifying tailored intervention strategies that promote digital well-being. The findings contribute to a growing body of research on the complex, multi-dimensional nature of technostress in digital environments and its implications for mental health, productivity, and social interaction.

### 1.1 Literature review

#### 1.1.1 SNS and technostress

Technostress, a modern form of stress induced by technology use, affects individuals across both professional and personal spheres [3,16]. It arises from the user's perceived lack of control over the technology environment, potentially threatening their well-being. Specific to SNS, platforms like Facebook introduce distinct technostressors—complexity, uncertainty, invasion, pattern, disclosure, and social overload—each contributing uniquely to the user's stress experience. These stressors range from the technical challenges and the invasion of personal life by SNS to the pressures of conforming to social norms and managing an overload of information [17].

#### 1.1.2 Characteristics of SNS

SNS offers unique features that profoundly shape social interaction in the digital age. According to Fox and Moreland [8], these platforms enable users to create personal profiles, connect with others, and navigate through these connections, enhancing direct and indirect social interactions. One of the fundamental advantages of SNS is their ability to foster a sense of connectedness by allowing users to seamlessly access and interact with their network. This access can sometimes lead to stress due to the visibility and permanence of online actions and information. For instance, posts or images that might be unflattering can remain indefinitely on one's profile, complicating the deletion process and affecting the individual's online social presence. The platforms facilitate a non-intrusive engagement with others' content, further contributing to the dynamics of social interaction and feedback through features like 'likes' and comments, which provide immediate social validation [8].

Moreover, the 'stimulus factors' on SNS—comprising the social, hedonic, and cognitive uses—drive user engagement by pursuing sociability, entertainment, and information, respectively. Studies have found a positive correlation between increased usage of SNS and elevated levels of technostress. This correlation indicates a complex relationship between user engagement and the stresses that come with digital connectivity [6,18].

#### 1.1.3 SNS technostress formation

The formation of technostress follows a natural progression beginning with the user's initial engagement with SNS. The platform's design features often facilitate this engagement, promoting regular usage and seamlessly integrating into daily routines. As users experience hedonic satisfaction and intensify their interaction with the platform, the actualization of IT affordances increases, accumulating engagement costs. Over time, these costs become burdensome, culminating in technostress when the demands of SNS usage exceed the user's adaptive capacities [2].

### 1.2 Key literature on technostress and SNS use

The rapid adoption of SNS has led to increased concerns about technostress and its effects on well-being. Previous systematic reviews (Table 1) provide varied perspectives on these concerns. For instance, Sanjeeva Kumar [19] examines technostress dimensions and management strategies while Bahamondes-Rosado, Cerdá-Suárez [20] focus on workplace technostress during the COVID-19 lockdown. Rademaker, Klingenberg and Süß [21] explore the role of leadership in mitigating technostress in organizational contexts. In contrast, studies by Hylkilä, Männikkö [14] and Wiwatkunupakarn, Pateekhum [22] examine how problematic SNS use affects social well-being in specific age groups, highlighting increased loneliness and isolation among both young adults and older adults.

**Table 1:** Summary of key literature on technostress and SNS use.

Category	Authors	Method	Themes	Research Gaps
Technostress	Nastjuk, Trang [12]	Meta-analysis	Technostress creators, outcomes	Need for a disaggregated view of stressors beyond work, emphasizing the SNS technostress-stressor relationship.
	Lin and Yu [13]	Systematic & Bibliometric	Technostress in higher education	Gaps in understanding stressors in hedonic SNS use contexts, lacking academic support structures found in educational settings.
	Sanjeeva Kumar [19]	Literature Review	Technostress dimensions, impacts, management	Need for in-depth analysis of technostress impacts on personal, hedonic SNS use where organizational support is absent.
	Bahamondes-Rosado, Cerdá-Suárez [20]	Systematic Review	Workplace technostress during COVID-19	Focused on workplace, limited non-work exploration voluntary SNS use contexts.
	Rademaker, Klingenberg and Süß [21]	Systematic Review	Leadership's influence on technostress	Insufficient focus on the effects of technostress in non-organizational, self-regulated environments like personal SNS use.
SNS Use	Astatke, Weng and Chen [24]	Literature Review	SNS impact on academic achievement	Limited focus on non-academic impacts of SNS use, particularly in terms of voluntary, hedonic stressors.
	Khalaf, Alubied [25]	Systematic Review	Social media's impact on mental health	Lack of empirical data on long-term mental health effects of SNS technostress specific to hedonic, personal use.
	Yu [26]	Systematic Review	SNS in educational context	Limited analysis on voluntary SNS use stress and its broader social implications beyond education.
	Hylkilä, Männikkö [14]	Systematic Review	Problematic SNS use, social well-being	Need for consistent definitions and clearer links between problematic SNS use and social well-being factors
	Wiwatkunupakarn, Pateekhum [22]	Systematic Review	SNS use, loneliness, and depression in older adults	Lack of experimental studies linking SNS use to social isolation reduction in older populations
	Akkaş and Turan [23]	Systematic Review	SNS use and life satisfaction	Lacks specific context of technostress. Need for further analysis on diverse demographic impacts on SNS life satisfaction outcomes
	Ansari, Iqbal [15]	Meta-analysis	Social media use and well-being	Broad overview, lacks specific context of technostress

While these reviews provide insights into specific aspects of SNS usage and technostress, gaps remain in understanding the full spectrum of SNS-induced technostress across non-work and personal contexts. For example, studies like Hylkilä, Männikkö [14]and Wiwatkunupakarn, Pateekhum [22] highlight social well-being challenges among specific age groups, but there is limited research comparing different demographics. Additionally, while reviews such as Ansari, Iqbal [15] examine social media's effects on well-being, they do not delve into the technostress mechanisms unique to SNS use. This review seeks to bridge these gaps by examining SNS technostress, immediate strains, and long-term outcomes of SNS-induced technostress across a broader population.

This paper, therefore, investigates technostress from the use of SNS in private lives, addressing the following research questions.

RQ1 Which studies have been conducted on personal technostress resulting from the use of SNS?

RQ2 What are the identified stressors, strains, and subsequent effects reported in the literature on personal technostress related to SNS?

RQ3 How do individuals cope with personal technostress arising from their use of SNS?

By exploring these dimensions, the study aims to deepen understanding of personal technology interactions, the stressors and coping mechanisms involved, and their implications for individual well-being.

## 2 Methodology

### 2.1 Protocol and registration

**Fig. 1:**
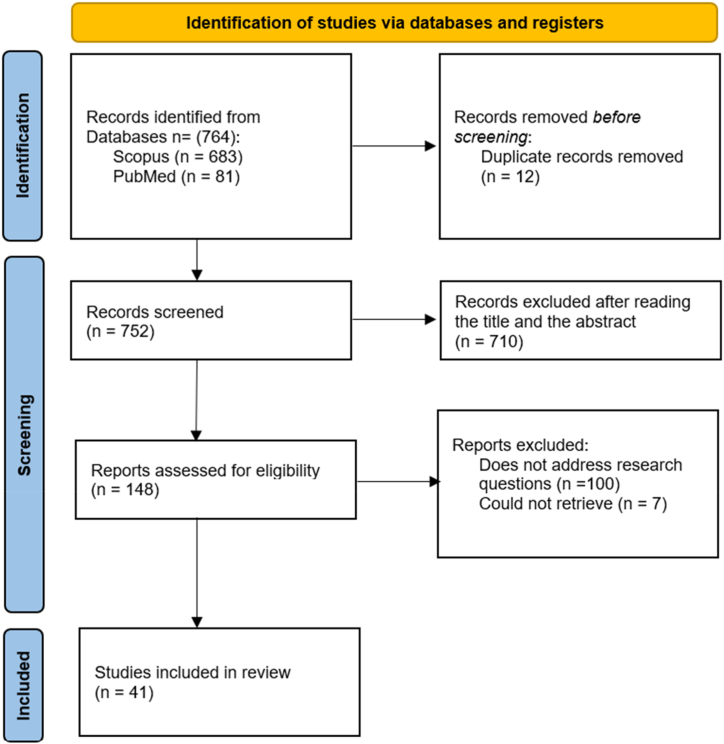
PRISMA flow chart of the results of the systematic review.

This systematic review was registered on the Open Science Framework (https://osf.io/7vezd/) [27] and conducted following the guidelines outlined in the PRISMA (Preferred Reporting Items for Systematic reviews and Meta-Analyses) 2020 declaration [28]. Fig. 1 depicts the flow chart outlining the process for selecting studies to be included in the systematic review.

### 2.2 Search strategy

**Fig. 2:**
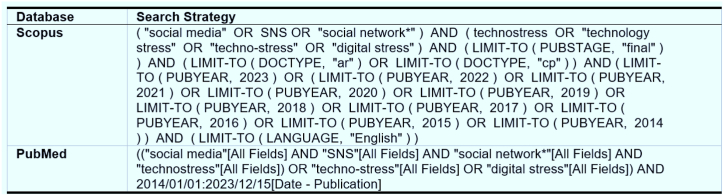
Search strategy for Scopus and PubMed databases.

The review utilized two well-regarded academic databases: PubMed and Scopus. Scopus, a prominent bibliographic database, serves as a primary resource for selecting journal articles [29]. Search queries for each database (Fig. 2) focused specifically on the titles and abstracts. The review employed an exhaustive search strategy incorporating field codes, Boolean operators, and proximity operators to ensure comprehensive searches. The operators 'AND' and 'OR' were used to refine the search: 'OR' included variations in spelling and synonyms, while 'AND' helped narrow the results to cover specific subjects more extensively. Tests were conducted on the target databases to evaluate their responsiveness to the study inquiry, with the initial search conducted on December 11, 2022, and updated on December 15, 2023.

### 2.3 Eligibility criteria

**Fig. 3:**
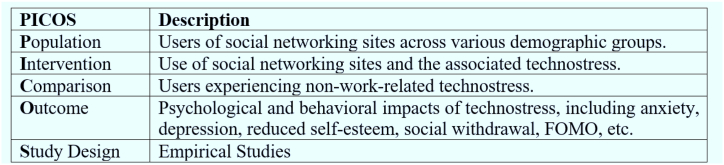
PICOS eligibility criteria.

The systematic searches adhered to the PICOS framework and were used as the criteria for including publications (Fig. 3), which comprise the following fundamental concepts: “Population, Intervention, Comparison, Outcome, and Study design”. This provides a structured approach to study selection [30,31]. The study's eligibility for inclusion was determined based on the following conditions.

### 2.4 Initial selection and data management

The search for relevant articles was limited to a specific time frame of publication, document type, and language (Table 2). Articles published between 2014 and 2023 were considered. Only English-language articles with empirical data were included in the evaluation to ensure accuracy and avoid translation difficulties, while non-English sources were excluded. Only peer-reviewed journal articles were included, while book series, chapters, and review articles were excluded. Table 2 outlines the additional inclusion and exclusion criteria. The authors evaluated the titles and abstracts of the papers to determine whether they met the criteria for inclusion in the study. An impartial eligibility assessment was conducted according to the established criteria, with resolutions reached through mutual agreement.

**Table 2:** The inclusion and exclusion criteria.

Criterion	Inclusion	Exclusion
Timeline	Between 2014 and 2023	<2014
Language	English	Non-English
Document Type	Journals and conference papers (research articles) with empirical data	Journal articles without empirical data, book series, chapters in books and systematic review papers

After the initial publication selection based on the inclusion criteria, the two authors used the EndNote citation management to filter out duplicates from Scopus and PubMed. The same authors then confirmed the accuracy of the extracted data. In cases of disagreement, pre-established criteria were used to resolve the issues. Any discrepancies during the data extraction process were resolved through discussion. The manuscript underwent multiple rounds of review, analysis, and documentation.

### 2.5 Coding and classification

A coding framework was developed based on key themes identified in the study results. This framework focused on stressors (e.g., social overload, information overload, communication overload), strains (e.g., emotional exhaustion, anxiety, depression), and outcomes (e.g., reduced SNS use, social withdrawal). Each eligible study was coded according to these variables to ensure structured and comparable analysis.

For each eligible study, data points relevant to the research questions were extracted, including identified stressors, strains, outcomes, and coping mechanisms. These elements were systematically coded in a standardized Excel sheet to organize studies into two main categories: SNS-Induced Technostress in Personal Contexts (covering stressors related to personal SNS use) and Psychological and Behavioral Outcomes of SNS Use (examining effects like emotional exhaustion and reduced SNS engagement due to stress).

Following coding, studies were synthesized using the "Stressor-Strain-Outcome" model, allowing us to examine relationships between identified stressors and psychological outcomes in personal SNS use. This approach enabled analysis of patterns across different demographics, identification of coping mechanisms, and examination of both immediate and long-term impacts of technostress on personal well-being.

### 2.6 Quality assessment

The methodological quality of the papers was independently assessed using the Mixed Methods Appraisal Tool (MMAT). This tool is designed to evaluate the risk of bias in studies employing quantitative, qualitative, and mixed-methods approaches [32]. This tool was particularly useful for appraising mixed-methods studies, which often involve a combination of data collection and analysis approaches. The MMAT checklist has two screening questions and 19 items across five methodological domains: qualitative research, randomized controlled trials (RCTs), non-randomized studies (NRS), quantitative descriptive studies, and mixed methods studies (MMS) [33,34].

In the assessment of mixed-methods studies, the MMAT requires separate evaluations of the qualitative and quantitative components, as well as an assessment of how well these components are integrated. For the qualitative component, the quality of the data was assessed by examining the clarity of research questions, the appropriateness of data collection methods (such as interviews and focus groups), and the thoroughness in reporting findings. Studies that lacked transparency in data collection procedures or provided insufficiently detailed findings were scored 0 on these criteria.

For the quantitative component, the robustness of sampling techniques, measurement reliability, and statistical rigor of the analysis were evaluated. Studies that used convenience sampling without justification or lacked valid and reliable measurement tools were noted for potential bias and received a score of 0 on the quantitative criteria. Additionally, the MMAT assesses the extent to which qualitative and quantitative findings are meaningfully integrated. This involves examining connections between the two components, such as whether qualitative findings are used to explain or expand on quantitative results. Studies that presented qualitative and quantitative results separately, without synthesis, were considered to have a higher risk of bias.

Two reviewers conducted the assessments independently, scoring each criterion as scored as1 (criteria met) or 0 (for criteria not met or unclear). After individual assessments, the reviewers discussed their evaluations to reconcile any discrepancies. For example, in cases where one reviewer identified a study lacking clear integration between qualitative and quantitative findings, both perspectives were discussed until a consensus was reached. The inter-rater reliability was strong, with a kappa (ĸ) value of 0.805, indicating a high level of inter-rater reliability in their MMAT assessments [35].

## 3 Results

During the initial search within the Scopus and PubMed databases, applying the exclusion criteria yielded 683 studies from Scopus and 81 from PubMed. From this combined pool, 12 duplicate studies were identified and removed. The remaining 752 studies underwent a preliminary review of titles and abstracts, excluding 710 studies that failed to meet the established eligibility criteria. This resulted in 148 studies being selected for a full-text review (Fig. 1).

After a detailed evaluation of these studies, 106 were further excluded. Among these, 100 did not adequately address the research questions, and seven were inaccessible. Following this extensive screening process, 41 studies met all inclusion criteria for this systematic review. Table 3 provides a comprehensive summary of these studies. The sample for this systematic review included twenty-three Quantitative Descriptive studies, one Quantitative Randomized Controlled Trial, eight Quantitative Non-Randomized studies, seven Qualitative studies, and two Mixed-methods studies (detailed references can be found in the MMAT supplementary file).

**Table 3:** Articles included in the analysis.

Paper ID	Title	Year	Journal	Theory	Participants
P1	Explaining the link between technostress and technology addiction for social networking sites: A study of distraction as a coping behavior	2020	Information Systems Journal	Feature-rich IT, the theory of technology frames, and distraction as a coping behaviour	EU Nations
Tarafdar, Maier [3]					
P2	How adolescents cope with technostress: A mixed-methods approach	2021	International Journal of Electronic Commerce	Transactional Theory of Stress	Germany
Schmidt, Frank and Gimpel [36]					
P3	Technostress and social networking services: Explaining users' concentration, sleep, identity, and social relation problems	2019	Information Systems Journal	Transactional Theory of Stress	Finland
Salo, Pirkkalainen and Koskelainen [37]					
P4	Empirical investigation of Facebook discontinues usage intentions based on SOR paradigm	2017	Computers in Human Behavior	Stimulus-organism-response (SOR) paradigm	China
Luqman, Cao [6]					
P5	The bittersweet escape to information technology: An investigation of the stress paradox of social network sites	2020	Information and Management	Stress–Release–Stress (SRS) process, regulatory focus theory (RFT) and conservation of resources theory (CORT)	North America
Cheikh-Ammar [4]					
P6	Stress caused by social media network applications and user responses	2017	Multimedia Tools Applications	Not specified	South Korea
Lim and Choi [38]					
P7	Online social media fatigue and psychological wellbeing—A study of compulsive use, fear of missing out, fatigue, anxiety and depression	2018	International Journal of Information Management	Stressor-strain-outcome (SSO) model	India
Dhir, Yossatorn [39]					
P8	Formation and Mitigation of Technostress in the Personal Use of IT	2022	MIS Quarterly	IT affordances and self-regulation	Finland
Salo, Pirkkalainen [2]					
P9	Digital stress within early adolescents' friendships – A focus group study from Belgium	2022	Telematics and Informatics	Socio-technical Systems Theory	Belgium
De Groote and Van Ouytsel [40]					
P10	Technology addictions and technostress: An examination of the U.S. and China.	2020	Journal of Organizational and End User Computing	Cognitive-Behavioural Model of Pathological Internet Use	USA, China
Brooks, Wang and Schneider [41]					
P11	Problematic social media use and associated consequences on academic performance decrement during	2022	Addictive Behaviors	Stressor-Strain-Outcome (SSO) model	Saudi Arabia
Ali Homaid [42]					
P12	Information and communication technology overload and social networking service fatigue: A stress perspective	2016	Computers in Human Behavior	Transactional Theory of Stress	South Korea
Lee, Son and Kim [43]					
P13	Exploring early adolescents' stressful IT use experiences	2023	Behavior & Information Technology	Techno-Bio-Psycho-Social theory	Finland
Mehtälä, Salo [16]					
P14	The dark side of social networking sites: An exploration of the relational and psychological stressors associated with Facebook use and affordances	2015	Computers in Human Behavior	Uses and Gratifications Theory	USA
Fox and Moreland [8]					
P15	The effects of technostress and switching stress on discontinued use of social networking services: a study of Facebook use	2015	Information Systems Journal	IS discontinuance	
Maier, Laumer [1]					
P16	Giving too much social support: social overload on social networking sites	2015	European Journal of Information Systems	Social support theory (SST)	Germany
Maier, Laumer [17]					
P17	Adverse consequences of emotional support seeking through social network sites in coping with stress from a global pandemic	2022	International Journal of Information Management	Transactional Theory of Stress and Coping	USA, India, Brazil, Canada, UK
Islam, Mäntymäki [44]					
P18	Exhaustion and dependency: A habituation–sensitization perspective on the duality of habit in social media use	2022	Information Technology & People	Dual process theory of habituation and sensitization	USA
Soror, Steelman and Turel [45]					
P19	Social media overload, exhaustion, and use discontinuance: Examining the effects of information overload, system feature overload, and social overload.	2020	Information Processing & Management	Stressor–strain–outcome framework IS discontinuance	global context via Amazon's Mechanical Turk
Fu, Li [46]					
P20	How social influence and personality affect users' social network fatigue and discontinuance behavior	2018	Aslib Journal of Information Management	Limited capacity model theory Uses and gratification theory	global context via Amazon's Mechanical Turk
Shokouhyar, Siadat and Razavi [47]					
P21	Do you get tired of socializing? An empirical explanation of discontinuous usage behaviour in social network services	2016	Information & Management	Stressor-strain-outcome framework	China
Zhang, Zhao [48]					
P22	A study on social overload in SNS: A perspective of Reactance Theory	2017	Pacific Asia Conference on Information Systems (PACIS) Proceedings	Reactance Theory	Japan
Lim, Park [49]					
P23	Does personal social media usage affect efficiency and well-being?	2015	Computers in Human Behavior	Distraction–Conflict Theory	USA
Brooks [18]					
P24	Digital stress: Adolescents' personal accounts	2015	New Media & Society	Uses and Gratifications Theory	USA
Weinstein and Selman [50]					
P25	Digital Stress over the life span: The effects of communication load and internet multitasking on perceived stress and psychological health impairments in a German probability sample	2017	Media Psychology	Transactional Theory of Stress	Germany
Reinecke, Aufenanger [51]					
P26	Adolescent digital stress: Frequencies, correlates, and longitudinal association with depressive symptoms	2022	Journal of Adolescent Health	Not specified	USA
Nick, Kilic [52]					
P27	Types of Social Media Use and Digital Stress in Early Adolescence	2023	The Journal of Early Adolescence	Transformation framework	UK
Winstone, Mars [53]					
P28	Short abstinence from online social networking sites reduces perceived stress, especially in excessive users	2018	Psychiatry Research	Not specified	USA
Turel, Cavagnaro and Meshi [54]					
P29	Facebook behaviors associated with diurnal cortisol in adolescents: Is befriending stressful?	2016	Psychoneuroendocrinology	Not specified	Canada
Morin-Major, Marin [55]					
P30	“I don't want to miss a thing”: Adolescents' fear of missing out and its relationship to adolescents' social needs, Facebook use, and Facebook related stress	2016	Computers in Human Behavior	Self-determination theory	Belgium
Beyens, Frison and Eggermont [56]					

Paper ID	Title	Year	Journal	Theory	Location
P31	Dark side of social media and academic performance of public sector schools students: Role of parental school support	2020	Journal of Public Affairs	Person-Environment Fit Model	Pakistan
Raza, Khan [57]					
P32	How technostress and self-control of social networking sites affect academic achievement and wellbeing	2022	Internet Research	Strength model of self-control	Ireland
Whelan, Golden and Tarafdar [58]					
P33	Influence of Technostress on Academic Performance of University Medicine Students in Peru during the COVID-19 Pandemic	2021	Sustainability	Stress–strain–outcome framework	Peru
Alvarez-Risco, Del-Aguila-Arcentales [59]					
P34	Privacy Management and Self-Disclosure on Social Network Sites: The Moderating Effects of Stress and Gender	2020	Journal of Computer-Mediated Communication	Communication Privacy Management (CPM) theory	Hongkong
Zhang and Fu [7]					
P35	Correlates of social media fatigue and academic performance decrement	2020	Information Technology & People	Stressor-strain-outcome framework	India
Malik, Dhir [60]					
P36	Effects of Personality Traits Concerning Media Use Decisions on Fear of Missing Out and Social Media Use Behavior	2022	Behavioral Sciences	Stressor-strain-outcome framework	Taiwan
Lin and Jian [61]					
P37	How Differential Dimensions of Social Media Overload Influences Young People's Fatigue and Negative Coping during Prolonged COVID-19 Pandemic? Insights from a Technostress Perspective	2023	Healthcare	Stressor-strain-outcome framework	China
Pang, Ji and Hu [62]					
P38	Reexploring Problematic Social Media Use and Its Relationship with Adolescent Mental Health. Findings from the “LifeOnSoMe”-Study	2023	Psychology Research and Behavior Management	Not specified	Norway
Finserås, Hjetland [63]					
P39 Lin, Fu and Zhou [9]	Unmasking the bright–dark duality of social media use on psychological well-being: A large-scale longitudinal study.	2023	Internet Research	Stressor-strain-outcome framework Social compensation hypothesis	China
P40	Elaboration of Social Media Performance Measures: From the Perspective of Social Media Discontinuance Behavior	2020	Sustainability	Not specified	South Korea
Kang, Zhang and Yoo [64]					
P41	Exploring the effect of overload on the discontinuous intention of social media users: An S-O-R perspective	2018	Computers in Human Behavior	S-O-R (Stimulus-Organism-Response) framework	China
Cao and Sun [65]					

### 3.1 Theoretical framework in SNS technostress studies

This review reveals a diverse application of the theoretical framework in SNS technostress studies, with the "Stressor-strain-outcome (SSO) framework" as the most frequently used model, appearing in ten articles. Researchers frequently integrate the SSO framework with other models to enrich their analyses. For instance, the "Transactional Model of Stress" was employed in five articles, highlighting its utility in deciphering stress dynamics. The "Uses and Gratifications Theory" and the concepts of "IS discontinuance" and the "Stimulus-Organism-Response (SOR) paradigm" were each explored in two articles, emphasizing their relevance in understanding stress dynamics. Additionally, the "Uses and Gratifications Theory" was referenced in two articles, aligning with user-centered research approaches. The concept of "IS discontinuance" and the "Stimulus-Organism-Response (SOR) paradigm" were each discussed in two articles, reflecting their significance in the context of technology usage and user response. Particularly, six studies lacked a specified theoretical framework, highlighting an opportunity for enhancing methodological transparency in future research. This eclectic use of theories emphasizes the interdisciplinary approach of technostress research, as scholars from different fields apply a variety of perspectives to dissect the complex interplay between technology use and its psychological effects.

### 3.2 Demographic analysis of SNS-induced technostress

From the 41 articles, an analysis was conducted to examine the demographic factors influencing SNS technostress. This analysis aimed to understand how age, gender, and cultural background shape the types and intensity of stressors and strains by users of SNS.

To distinguish between stressors, strains, and outcomes, the Stressor-Strain-Outcome (SSO) model [66] was used as a framework for understanding how external stressors lead to specific psychological and behavioral responses. The SSO model is the most frequently applied framework in the 41 reviewed articles. In Table 4, strains and outcomes are combined as a unified category that aligns with findings in digital stress research, which indicate immediate stress responses and long-term impacts often overlap [44,67].

**Table 4:** Summary table of demographic variables, stressors, and strains and outcomes.

Paper ID	Age Group	Demographic Group	Gender	Cultural Background	Primary Stressors	Primary Strains and Outcomes
P1	18+	General Users (avg age 35.81)	Not specified	Western	Social overload, invasion, uncertainty, complexity	SNS addiction, fatigue, decreased satisfaction
P2	10–17	Adolescents	Female	German	Higher privacy concerns, disclosure	Higher anxiety, fatigue, difficulty in regulating ICT usage
P2	10–17	Adolescents	Male	German	Lower privacy concerns, social overload	Less anxiety, moderate fatigue, increased ICT regulation challenges
P3	20–80	General Users	Not specified	European	Identity issues, social relation problems	Concentration problems, sleep issues, burnout
P4	19–44	Not specified	Not specified	Chinese	Social overload, hedonic use	Intention to quit, SNS exhaustion
P5	Not specified	Not specified	Not specified	Canadian	Privacy threats, social demands, overload	Stress relief paradox, addiction, exhaustion
P6	18–25	Not specified	Not specified	South Korean	Social comparison, privacy, overload	Emotional exhaustion, intention to switch, resistance
P7	12–18	Adolescents	Not specified	Indian	Compulsive use, fear of missing out	Fatigue, anxiety, depression
P8	20–80	Adults	Not specified	Finnish	Dependency, privacy concerns	Cognitive strain, burnout, need for self-regulation
P9	13–16	Adolescents	Female	Belgian	Higher fear of missing out, availability stress	Higher social anxiety, increased friendship tension
P9	13–16	Adolescents	Male	Belgian	Moderate fear of missing out, lower availability stress	Lower social anxiety, moderate friendship tension
P10	18+	Not specified	Not specified	U.S.	High social media addiction, technostress	Increased anxiety, reduced productivity
P10	18+	Not specified	Not specified	Chinese	Moderate social media addiction, technostress	Lower anxiety, higher perceived social pressure
P11	21–23	Undergraduate students	Not specified	Saudi Arabian	Problematic social media use, technostress	Academic performance decrement, exhaustion
P12	Not specified	Adults	Not specified	South Korean	Information, communication, system feature overload	SNS fatigue, psychological and physical strain
P13	13–15	Adolescents	Not specified	Finnish	Notification-driven stress, online behavior challenges	Physical strain (headache), emotional stress
P14	19–52	Adults	Not specified	U.S.	Social comparison, jealousy, privacy management	Relationship conflict, psychological distress
P15	16–42	Adults	Not specified	German	Technostress, switching stress	Discontinued usage intentions, SNS exhaustion
P16	Not specified	Adults	Not specified	German	Social overload from social support	SNS exhaustion, decreased satisfaction, intention to reduce SNS use
P17	Not specified	Not specified	Not specified	Global	COVID-19 obsession, emotional support seeking	SNS exhaustion, intention to reduce SNS use
P18	18–70	Adults	Not specified	U.S.	SMU dependency, habitual use	SMU-related exhaustion, dependency
P19	Not specified	Not specified	Not specified	Chinese	System feature overload, information overload	Social media fatigue, discontinuance intention
P20	18+	Not specified	Not specified	Iranian	Social influence, personality-related social overload	Preference for controlled SNS usage
P21	18–35	Not specified	Not specified	Chinese	System feature overload, information overload	Social network fatigue, intention to discontinue SNS use
P22	20–29	Not specified	Not specified	Japanese	Social overload, threat to usage freedom	Reactance, discontinuation of SNS usage
P23	18–29	Not specified	Not specified	U.S.	Personal use during professional time, distraction	Technostress, lower task performance, reduced well-being
P24	Not specified	Adolescents	Not specified	U.S.	Harassment, public shaming, relational tension	Emotional distress, anxiety, relational strain
P25	14–85	Not specified	Mixed	German	Communication load, internet multitasking	Burnout, depression, anxiety
P26	Not specified	Adolescents	Mixed	U.S.	Social media demands, peer judgment	Depressive symptoms, increased digital stress
P27	13–14	Early adolescents	Not specified	U.K.	Peer pressure, social comparison	Digital guilt, fear of negative evaluation, identity stress
P28	20–38	Adults	Not specified	U.S.	Habitual use, SNS dependency	Reduced perceived stress with SNS abstinence
P29	12–17	Adolescents	Male	Canadian	Social validation, fewer peer interactions stress	Moderate cortisol response, lower stress than females
P29	12–17	Adolescents	Female	Canadian	Peer interactions, social validation stress	Elevated cortisol, higher emotional stress
P30	Not specified	Adolescents	Not specified	Belgian	Fear of missing out (FoMO), need for popularity	Facebook-related stress, anxiety
P31	Not specified	Adolescents Public school students	Not specified	Pakistani	Cyberbullying, media multitasking	Lower academic performance, technostress
P32	18+	University students	Not specified	Irish	Social overload, reduced self-control on SNS	Academic achievement decline, reduced well-being
P33	18–26	Medical students	Not specified	Peruvian	Communication overload, social overload	Technostress, mental exhaustion, reduced academic performance
P34	18–25	University students	Female	Hong Kong	Higher privacy concerns under stress	Decreased self-disclosure, increased emotional strain
P34	18–25	University students	Male	Hong Kong	Moderate privacy concerns under stress	Lesser impact on self-disclosure, lower stress-induced privacy management challenges
P35	18–27	Young adults	Not specified	Indian	Social comparison, fear of missing out (FoMO)	Social media fatigue, decline in academic performance
P36	Not specified	Young adults General SNS users	Not specified	Taiwanese	Social comparison, regret tendency	Compulsive SNS use, emotional distress
P37	Not specified	Young adults	Mixed	Chinese	Information and communication overload	Social media fatigue, negative coping responses during COVID-19 pandemic
P38	Not specified	Adolescents	Female	Norwegian	Subjective overuse, social obligations	Higher anxiety, emotional strain, reduced well-being
P39	Not specified	Adolescents	Male	Norwegian	Social obligations, source of concern	Moderate anxiety, minimal reduction in well-being
P40	20+	Not specified	Not specified	South Korean	Social media fatigue, emotional overload	Discontinuance intention, social withdrawal
P41	20–31	Adults	Not specified	Chinese	Information, communication, social overload	SNS exhaustion, regret, discontinuous

### 3.3 Age-based differences and primary stressors

**Fig. 4:**
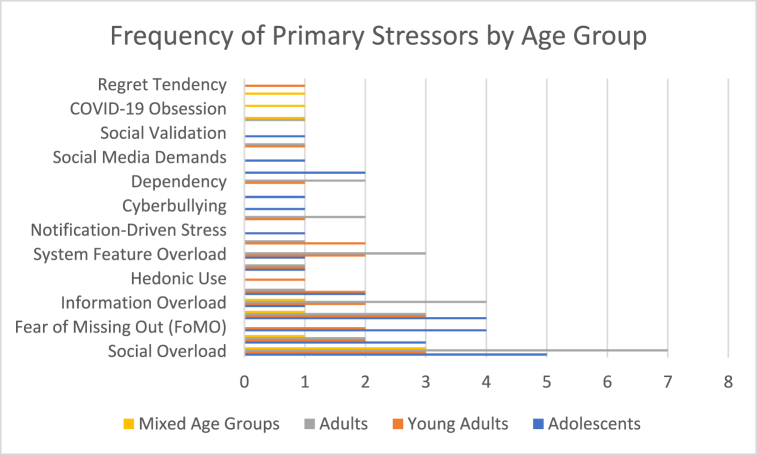
Primary stressors by age group.

The studies encompassed a wide range of age groups, categorized as adolescents (up to 18 years old), young adults (18–29 years old), adults (30 years and above), and mixed age groups (Fig. 4). The primary stressors varied significantly across these age categories.

#### 3.3.1 Adolescents

Adolescents are among the most active SNS users and often face unique stressors due to developmental and social factors. Schmidt, Frank and Gimpel [36] investigated German adolescents aged 10–17 and found that female adolescents exhibited higher privacy concerns and disclosure-related stressors, leading to increased anxiety and fatigue, as well as greater difficulty in regulating ICT usage. Male adolescents in the same age group reported lower privacy concerns but experienced social overload, resulting in moderate fatigue and challenges in ICT regulation.

Dhir, Yossatorn [39] studied Indian adolescents aged 12–18 and identified compulsive use and fear of missing out (FoMO) as primary stressors, which led to fatigue, anxiety, and depression. Similarly, De Groote and Van Ouytsel [40] explored Belgian adolescents aged 13–16 and observed that females experienced higher FoMO and availability stress, correlating with higher social anxiety and increased friendship tension. Males reported moderate FoMO and lower availability stress, with correspondingly lower social anxiety.

Winstone, Mars [53] found that early adolescents in the U.K. aged 13–14 faced peer pressure and social comparison as stressors, leading to digital guilt, fear of negative evaluation, and identity stress. Mehtälä, Salo [16] reported that Finnish adolescents aged 13–15 experienced notification-driven stress and online behavior challenges, resulting in physical strain like headaches and emotional stress.

#### 3.3.2 Young adults

Among young adults, Lim and Choi [38] focused on South Korean university students aged 18–25, identifying social comparison, privacy concerns, and overload as significant stressors leading to emotional exhaustion, intention to switch platforms, and resistance to SNS usage. Zhang and Fu [7] examined Hong Kong university students aged 18–25, finding that female students had higher privacy concerns under stress, leading to decreased self-disclosure and increased emotional strain, whereas male students showed moderate privacy concerns and lesser impact on self-disclosure.

Malik, Dhir [60] studied Indian young adults aged 18–27 and found that social comparison and FoMO were significant stressors leading to social media fatigue and a decline in academic performance. Pang, Ji and Hu [62] observed that Chinese young adults experienced information and communication overload, resulting in social media fatigue and negative coping responses during the COVID-19 pandemic.

#### 3.3.3 Adults

Adults also experience technostress differently. Tarafdar, Maier [3]studied Western users with an average age of 35.81 years, identifying social overload, invasion, uncertainty, and complexity as stressors that led to SNS addiction, fatigue, and decreased satisfaction. Salo, Pirkkalainen and Koskelainen [37] investigated European adults aged 20–80 and found that identity issues and social relation problems were primary stressors resulting in concentration problems, sleep issues, and burnout.

Fox and Moreland [8] examined U.S. adults aged 19–52 and identified social comparison, jealousy, and privacy management as stressors leading to relationship conflict and psychological distress. Reinecke, Aufenanger [51] studied German users found that communication load and internet multitasking led to burnout, depression, and anxiety.

#### 3.3.4 Mixed groups

In the mixed age groups, studies highlighted general stressors like identity issues and technostress, which affect users across different age ranges [37,44].

### 3.4 Gender-based variations in technostress experience

**Fig. 5a:**
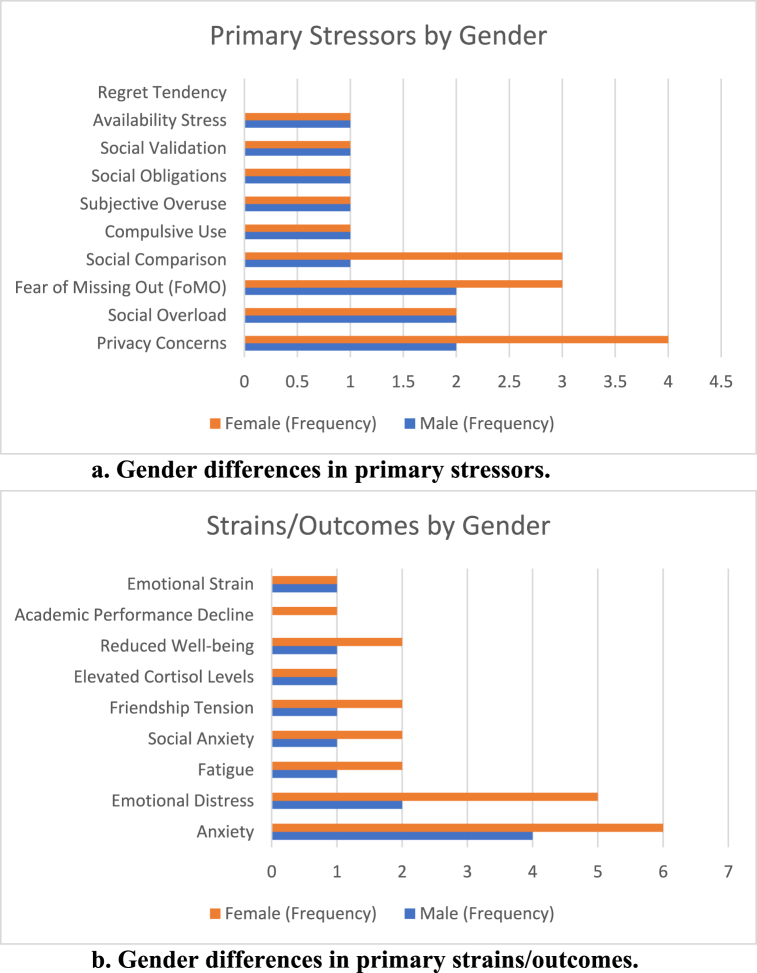
Gender differences in primary stressors, strains/outcomes. Fig. 5a Gender differences in primary stressors. Fig. 5b Gender differences in primary strains/outcomes.

Gender differences influence the experience of technostress on SNS. Female users often report higher levels of certain stressors (Fig. 5a) and strains (Fig. 5b) than male users.

Schmidt, Frank and Gimpel [36] found that German female adolescents had higher privacy concerns and disclosure stressors, leading to increased anxiety and fatigue. In contrast, male adolescents reported lower privacy concerns but experienced social overload. De Groote and Van Ouytsel [40] observed that Belgian female adolescents experienced higher FoMO and availability stress, correlating with higher social anxiety and friendship tension.

Morin-Major, Marin [55] studied Canadian adolescents aged 12–17 and found that females experienced elevated cortisol levels and higher emotional stress due to peer interactions and social validation stress, while males had a moderate cortisol response and lower stress levels. Lin, Fu and Zhou [9] investigated Norwegian adolescents and found that females experienced higher anxiety, emotional strain, and reduced well-being due to subjective overuse and social obligations, while males reported moderate anxiety with minimal reduction in well-being.

Zhang and Fu [7] reported that Hong Kong female university students had higher privacy concerns under stress, leading to decreased self-disclosure and increased emotional strain, whereas male students were less impacted. Lin and Jian [61] found that Taiwanese young adults experienced social comparison and regret tendency as stressors, leading to compulsive SNS use and emotional distress, with females reporting higher levels of distress.

### 3.5 Cultural background and technostress

**Fig. 6:**
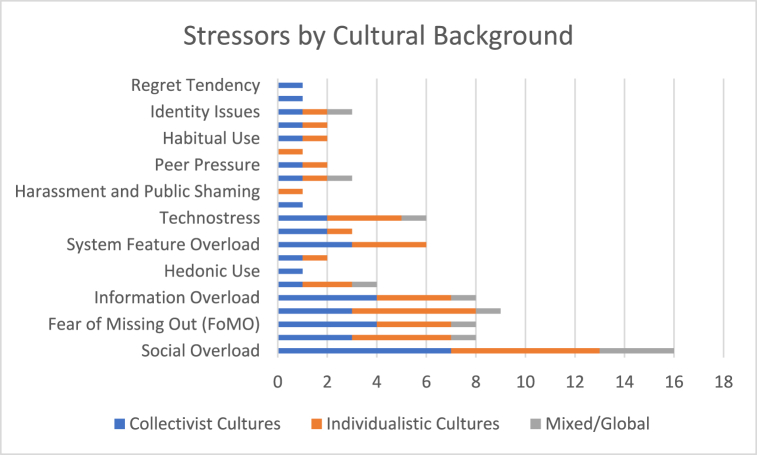
Primary stressors by cultural background.

Cultural context also influenced the nature of stressors experienced by SNS users. The studies categorized cultural backgrounds into collectivist cultures (e.g., China, South Korea, India), individualistic cultures (e.g., the United States, United Kingdom, Germany), and mixed/global contexts (Fig. 6).

#### 3.5.1 Collectivist cultures

In collectivist cultures like China and South Korea, communal relationships and interdependence are core values that shape users' experiences on social media platforms. Social factors such as social overload and hedonic use are prominent stressors in these societies. For instance, Luqman, Cao [6] found that Chinese users aged 19–44 experienced social overload and hedonic use as primary stressors, leading to intentions to quit and SNS exhaustion. Similarly, Lim and Choi [38] identified social comparison, privacy concerns, and overload as significant stressors among South Korean users, resulting in emotional exhaustion.

Zhang and Fu [7] observed that Chinese users faced system feature overload and information overload, which led to social media fatigue and discontinuance intention. During the COVID-19 pandemic, Pang, Ji and Hu [62] reported that Chinese young adults experienced heightened information and communication overload, resulting in social media fatigue and negative coping responses. Despite the higher levels of technostress, the communal nature of collectivist societies can mitigate feelings of loneliness, contrasting sharply with the individualistic stress outcomes observed in the United States [41]. The ease of use and the role of social media in maintaining interpersonal connections are heavily valued in Eastern societies, reducing the technostress associated with its use and underscoring a cultural preference for stress-free social interactions [41].

#### 3.5.2 Individualistic cultures

Conversely, in individualistic cultures like the United States, where personal productivity and success are highly valued, users experiencing SNS addiction encounter heightened levels of technostress due to the conflict between compulsive social media use and societal expectations. Turel, Cavagnaro and Meshi [54] noted that U.S. users face increased technostress stemming from this clash between excessive SNS use and the cultural emphasis on productivity. Tarafdar, Maier [3] identified social overload, invasion, uncertainty, and complexity as primary stressors among Western users, leading to SNS addiction and decreased satisfaction.

Brooks, Wang and Schneider [41] compared U.S. and Chinese users and found that U.S. users exhibited high social media addiction and technostress, resulting in increased anxiety and reduced productivity, whereas Chinese users had moderate social media addiction but experienced higher perceived social pressure. Fox and Moreland [8] examined U.S. adults and identified social comparison, jealousy, and privacy management as stressors that led to relationship conflict and psychological distress. Whelan, Golden and Tarafdar [58] observed that Irish university students faced social overload and reduced self-control on SNS, leading to academic achievement decline and reduced well-being. In Western contexts, the emphasis on the productivity-enhancing potential of social media can exacerbate technostress when using these platforms conflicts with achieving personal goals [41].

#### 3.5.3 European contexts

European studies further highlight cultural influences on technostress. Salo, Pirkkalainen [2], Salo, Pirkkalainen and Koskelainen [37] examined Finnish and European users, finding that identity issues, social relation problems, and dependency were significant stressors leading to cognitive strain, burnout, and the need for self-regulation. De Groote and Van Ouytsel [40] reported that Belgian adolescents experienced FoMO and availability stress, correlating with higher social anxiety and friendship tension. These studies suggest that even within individualistic cultures, regional cultural norms and values can influence how technostress manifests.

#### 3.5.4 Mixed/global studies

In mixed/global studies highlighted universal stressors like social overload and identity issues that transcend cultural boundaries [44].

### 3.6 SNS-technostress and impact on academic performance

**Fig. 7:**
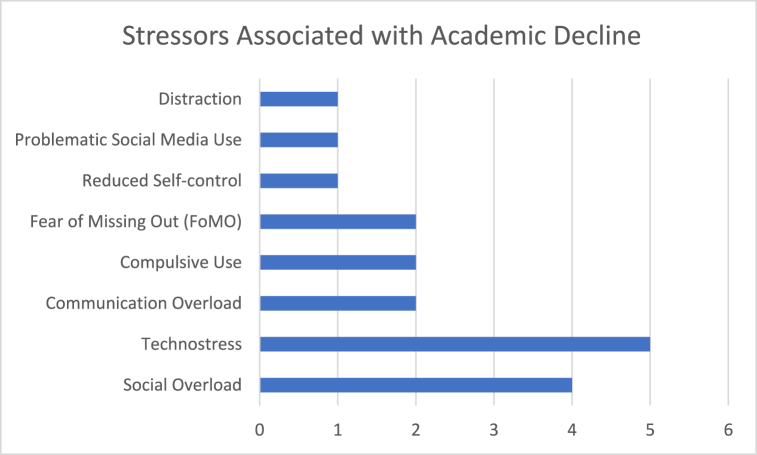
Stressors associated with academic decline.

Several studies highlighted the adverse effects of SNS-induced technostress on academic performance (Fig. 7). The adverse effects of problematic SNS usage also extend into the academic realm, where excessive engagement with social media correlates with poor academic performance. Technostress further diminishes their mental capacity to focus on academic tasks, leading to a decline in academic performance [58,60]. Studies indicate that the persistent distraction and cognitive overload associated with frequent SNS use can significantly detract from students' ability to achieve educational goals [60,65]. Compulsive use and FoMO contributed to distractions and reduced self-control, further impacting academic achievements [39,57]. Whelan, Golden and Tarafdar [58] highlighted that social media overload negatively impacts students' psychological well-being, exacerbating stress and reducing overall life satisfaction. These findings underscore the need for targeted strategies that address the root causes of SNS-induced stress and exhaustion to preserve the academic and psychological well-being of students.

### 3.7 Conceptualization of technostress in SNS situations

RQ1: Which studies have been conducted on personal technostress resulting from the use of SNS?

Driven by the widespread use of SNS in contemporary culture, there has been a significant rise in research on stress within SNS environments. This study categorizes these investigations into three major themes, each reflecting different aspects of stress, strains, and outcomes in SNS situations.

#### 3.7.1 Theme 1: “technostress as triggering stressors”

Research focusing on the first theme conceptualizes stress regarding stress producers. The effects of single or several stressors (collectively called technostress) on significant dependent variables are explored. Using this concept of technostress, researchers investigated non-work-related SNS and discovered that stress induced by the use of personal SNS such as Facebook is associated with addiction [3,40] and is negatively associated with happiness [18] and self-esteem [53]. Technostress is often driven by excessive usage [6] and personality factors [47,61]. Studies indicate that these stressors can lead to addiction and that users may cope with technostress by shifting focus to less stressful SNS features [3]. This theme aligns with the SSO model by recognizing stressors as the initial triggers that may result in further strains and long-term psychological outcomes.

#### 3.7.2 Theme 2: “technostress as stressor-strain interaction”

In this theme, technostress is defined by both its stressors and the immediate strains they produce. Researchers have identified fatigue and emotional exhaustion as common strains arising directly from SNS pressures, where these strains act as psychological and behavioral response to constant engagement and information overload [2,39,43]. Exhaustion is also referred to simply as psychological strain [4,16,38], is usually believed to result in dissatisfaction with SNSs and may prompt users to reduce their engagement, switch to alternative platforms, or even discontinue use altogether as coping strategies [[46], [47], [48]]. However, switching to a different SNS has also been shown to introduce new stressors; thus, it is possible that it may mitigate the effects of technostress [49].

The long-term outcomes of technostress include decreased performance and negative academic impacts, which are considered enduring psychological and behavioral consequences of sustained exposure to strains like fatigue and exhaustion [42,59,65]. This theme demonstrates the role of strains as immediate reactions to stressors, with prolonged strain exposure leading to significant, long-term outcomes.

#### 3.7.3 Theme 3: “Stress as a general perception and well-being indicator”

The third theme of research is neither centered on stress nor does it study its characteristics. The third research topic neither focuses on stress nor examines its characteristics. Instead, stress is studied as one of the multiple individual well-being indicators. As an emotional reaction to SNS usage (other reactions include “mood, perceived social support, life satisfaction, despair, and loneliness”), stress is considered a general impression that is impacted by elements relating to the SNS structure and size-related factors [4]. For instance, it is hypothesized that a high stress level would diminish the link between privacy concerns and the quantity, closeness, sincerity, and intention of self-disclosure on SNSs [53].

### 3.8 Identifying stressors, strains, and outcomes in SNS-induced technostress

RQ2: What are the identified stressors, strains, and subsequent effects reported in the literature on personal technostress related to SNS?

As mentioned, to distinguish between stressors, strains, and outcomes of SNS-induced technostress, the SSO model by Koeske and Koeske [66] was used. In this model.

• *Stressors* refer to SNS elements like information overload that directly induce stress.
• *Strains* are immediate psychological and behavioral responses to these stressors, such as perceived stress and burnout.
• *Outcomes* represent enduring psychological consequences resulting from prolonged exposure to strains, such as depression and anxiety.

While strains and outcomes may overlap in digital stress contexts, separating them in this analysis provides a clearer view of the immediate versus cumulative impacts of SNS stressors. This separation helps capture both the direct effects of SNS stressors on user experience and the potential long-term consequences on well-being, allowing for a more comprehensive understanding of SNS-induced technostress.

#### 3.8.1 Techno-social stressors: causes of SNS stress

The widespread adoption of SNS has introduced a spectrum of techno-social stressors that impact users' mental health and social interactions. These stressors are broadly classified into three categories: technological, behavioral-technological, and social stressors, each contributing uniquely to the overall technostress experienced by users (Table 5).

**Table 5:** SNS stressors.

Classification	SNS-stressors	Paper ID
Techno-stressor	Complexity	P1, P2, P10, P15, P31, P32, P40
	Uncertainty	P1, P2, P15, P32
	Invasion	P1, P2, P8, P10, P15, P31, P32, P36, P40
	Managing inappropriate or annoying content	P14
	Exposure to harmful content	P26
	Nomophobia	P39
Behavioral-Technological Stressors	Excessive SNS use	P27, P30, P4, P11
	Excessive (hedonic/cognitive) use	
	Compulsive SNS use	P9
	Excessive social use	P7
	Dependency/SNS overdependency	P3, P8, P13
	Passive social media use	P26
	Time-wasting and digital guilt	
Social-stressors	Pattern	P1, P2, P15, P32
	Techno-overload/System feature overload	P3, P36
	Information overload	P12, P19, P20, P21, P37
	Social overload	P12, P13, P19, P20, P21, P31, P37, P40
	Communication overload	P12, P33, P35, P37
	Social overload	P1, P2, P3, P4, P5, P6, P10, P15, P18, P19, P20, P21, P22, P31, P32, P33, P40, P41
	Disclosure	P1, P2, P15, P32
	Self-Disclosure	P31, P34, P35, P40
	Fear of Missing Out (FOMO)	P7, P9, P25, P29, P31, P35, P40
	Online Vigilance	P9
	Social Pressure	P2, P25
	Life comparison discrepancy	P3, P13
	Social comparison and jealousy	P6, P14, P35, P36
	Insecurity	P2
	Expectations of perfection and sexualization	P26
	Online discussion conflict	P3, P13
	Relationship tension and conflict	P14
	Privacy (risks/threats/concerns)	P5, P6, P8, P34, P35, P26, P3, P13, P14, P30
	Biased opinion	P6
	Availability (expectations)	P9; P26
	Approval anxiety fear of negative evaluation	P9 P26
	Emotional support seeking (through SNS)	P17

##### 3.8.1.1 Technological stressors

These stressors derive from the inherent design and operational characteristics of SNS platforms, including complexity, uncertainty, and invasiveness. The complex interfaces and multifunctional options of SNS platforms can overwhelm users, particularly those not technologically savvy [17]. Frequent updates further contribute to this stress by changing user interfaces and functionalities, forcing users to continually adapt their habits and strategies [36]. This ongoing adjustment can lead to uncertainty and frustration, impacting users' ability to utilize the platforms effectively [68].

SNS platforms often push frequent notifications and alerts, interrupting daily life and encroaching on personal time [41]. Furthermore, the extensive data collection practices by these platforms can lead to feelings of surveillance and a significant reduction in personal privacy, increasing stress and discomfort among users [61].

##### 3.8.1.2 Behavioral-technological stressors

This category highlights the intersection of user behavior with technological features, including compulsive and excessive use of SNS, which often leads to significant stress impacting mental health and productivity. Compulsive SNS use and excessive social use, driven by hedonic (pleasure-seeking) and cognitive (information-seeking) motivations, can lead to technostress. This usage often feels out of the user's control and can negatively impact personal responsibilities and relationships [8]. The addictive nature of these platforms, designed to keep users engaged for long periods, exacerbates this stress, leading to significant impacts on mental health and productivity [50].

##### 3.8.1.3 Social stressors

Rooted in user interactions and exposure dynamics, social stressors on SNS such as Facebook significantly affect users' social well-being and mental health. These stressors include social overload, online conflicts, and privacy concerns. For instance, the complex social dynamics of these platforms can exacerbate feelings of inadequacy and jealousy, as users often compare their lives with idealized portrayals online [8,38]. Such comparisons can lead to persistent stress and affect mental health. Furthermore, users face an overwhelming influx of social requests and experience online conflicts, which refer to disagreements on these platforms [59]. The act of self-disclosure, involving sharing personal thoughts, feelings, and experiences, raises significant privacy concerns and vulnerability to negative feedback [64].

Social pressure to meet others' expectations further compounds these stressors, as it can lead to both passive and active distress. Passive use of SNS, such as browsing without meaningful interaction, often results in time-wasting and digital guilt, whereas active use, like social media broadcasting, can provoke stress due to fears of negative evaluation and privacy risks [53]. Moreover, the constant accessibility of SNS through mobile apps leads to fears of missing out (FoMO) and nomophobia—the anxiety of being without one's smartphone—reflecting the deep dependency developed on these technologies [39,42]. This constant connectivity expectation contributes to availability anxiety, demanding immediate responses and constant online presence [40].

Research by Turel, Cavagnaro and Meshi [54] highlights the diverse experiences of adolescents with digital stress, revealing that individual usage patterns can significantly influence well-being. This variation points to the necessity for interventions specifically designed to address the unique stressors encountered by different user demographics. Tailoring these interventions is crucial to effectively mitigate the diverse effects of digital stress on young users.

#### 3.8.2 Strains: immediate consequences of SNS-induced technostress

Technostress from SNS use leads to various immediate consequences, or strains, impacting users’ psychological, behavioral, and physical well-being (Table 6).

**Table 6:** Immediate strains resulting from SNS-induced technostress.

Classification	Strains	Paper ID
Psychological strain	SNS-exhaustion emotional exhaustion	P4, P5, P6, P8, P11, P13, P15, P16, P17, P30, P33
	SNS (dis) satisfaction	P16, P22
	Identity problems Social relation problems	P3
	Jealousy and other negative emotions	P14
Behavioral	Media-induced meal skipping and fast eating	P29
	Time management problems	P8
	Resistance	P6
Physical wellbeing	SNS fatigue	P7, P8, P12, P20, P21, P31, P34, P37
	Concentration Problems	P3, P8
	Headache	P13
	High level of cortisol	P28

##### 3.8.2.1 Psychological strains

Psychological strains encompass a range of emotional responses caused by ongoing SNS interaction. One prominent form is SNS exhaustion, reflecting the significant emotional toll from constant connectivity. This condition is marked by exhaustion, which can escalate to more severe psychological issues like depression, anxiety, and burnout, substantially impacting mental health [17,39,51,53,55]. The information and communication overload contribute to cognitive saturation, where the sheer volume of incoming information becomes difficult to process [43,51]. Social relationship problems and jealousy stemming from online comparisons and interactions often lead to negative emotional experiences [8,37].

##### 3.8.2.2 Behavioral strains

Behavioral strains refer to changes in user habits driven by SNS-induced stress. The influence of SNS on meal routines often leads to meal skipping or fast eating as users become distracted by continuous media consumption and notifications. Prioritizing updates, engaging with content, or responding to messages over proper mealtime practices results in rushed or missed meals, demonstrating a compulsion to stay connected even at the expense of health and well-being [1,6].

The SNS usage frequently disrupts effective time management. The constant influx of updates, notifications, and social pressures diverts user attention, making it challenging to allocate time effectively for other tasks and responsibilities [4]. This disruption can lead to procrastination, reduced productivity, and an inability to set healthy boundaries, as users struggle to balance SNS engagement with daily activities [41].

To cope with these stressors, users often exhibit resistance behaviors such as limiting, reducing, or completely ceasing SNS usage—a coping response known as discontinuance of usage [1,3,45]. Discontinuance reflects a conscious effort by users to regain control over their time and mental resources by curbing engagement with SNS when stress levels become overwhelming.

##### 3.8.2.3 Physical strains

Physical strains directly result from excessive SNS use (Table 6), including SNS fatigue and sleep problems that cause physical tiredness and disrupt normal sleep patterns, indicative of the physiological toll of prolonged screen time [2,39,43]. Other symptoms, such as headaches and elevated cortisol levels, signal significant physical stress responses, further highlighting the comprehensive strain imposed by habitual SNS use [16,45].

#### 3.8.3 Outcome: long-term consequences of strain exposure

Long-term exposure to SNS-induced technostress results in various outcomes, primarily psychological, behavioral, and physical (Table 7).

**Table 7:** Long-term outcomes of SNS-induced technostress.

Classification	Outcomes	Paper ID
Psychological	Depression	P7, P25, P29, P39
	Anxiety	P7, P14, P25, P29, P40
	Burnout	P25
	Lower level of happiness/Loneliness	P23, P39
	Poor self-esteem	P26
	Regret	P36, P41
Behavioral	Discontinuance of use	P4, P15, P16, P18, P19, P20, P21, P22
	Reduce/Decrease SNS use	P17
	Taking breaks from SNS	P15
	Switch intention	P6
	SNS Addiction	P1, P9, P10
Physical wellbeing	Sleep problems	P29

##### 3.8.3.1 Psychological

Prolonged SNS technostress can lead to chronic mental health issues, including depression, anxiety, loneliness, and reduced self-esteem. These conditions reflect the enduring effects of stress and can severely impair emotional well-being over time [39,53,55]. Additionally, users frequently report regret about online actions or disclosures, especially if they feel they have over-shared or misrepresented themselves [52,56].

##### 3.8.3.2 Behavioral

Long-term technostress leads many users to reduce their SNS engagement or discontinue use altogether. The complexity, perceived invasiveness, and general stress associated with SNS often heighten the desire to disengage. In contrast, users who habitually engage or derive enjoyment from SNS use may experience reduced intentions to discontinue, despite the presence of stressors [1,45]. Switching intentions—the desire to move to different platforms or take breaks—are also common as users attempt to manage stress levels [6,65].

In discontinuance, research points to a complex interaction between user behavior, stress, fatigue, and decisions to continue or disengage from SNS use. Complexity, uncertainty, and perceived invasiveness intensify users' intentions to discontinue use. Conversely, the stresses of switching and exhaustion might lessen these intentions. Empirical studies also suggest that enjoyment with SNS may paradoxically decrease users’ desire to discontinue, despite the presence of stressors [1,45].

##### 3.8.3.3 Physical well-being

Persistent sleep problems are among the most common physical outcomes of long-term SNS technostress. Sleep disturbances result from compulsive checking, high engagement levels, and frequent SNS interruptions, all of which interfere with regular sleep patterns and exacerbate other physical and mental health issues [41].

### 3.9 Coping: to mitigate the negative impact of technostress

RQ3: How do individuals cope with personal technostress arising from their use of SNS?

Coping with the negative impact of technostress, particularly arising from SNS use, encompasses a range of strategies to manage, reduce, or tolerate the stress associated with digital interactions. Individuals deploy various coping mechanisms based on their available resources, such as social support, positive beliefs, and self-efficacy, influencing their choice between approach and avoidance coping strategies [49].

#### 3.9.1 Approach coping strategies

These involve actively managing environmental and emotional stress through active coping, cognitive behavioral techniques, and problem-focused coping. Such strategies are noted for their effectiveness in moderating the relationship between SNS stress and outcomes like emotional tiredness, the intention to switch platforms, and enhancing resistance to stressors [38].

#### 3.9.2 Avoidance coping strategies

When individuals perceive their ability to manage SNS-induced stress as inadequate, these strategies may include diverting attention to other activities, passively tolerating criticism, or venting emotions to momentarily forget problems. The efficacy of these strategies can depend significantly on the individual's perception of stress as either a challenge or a threat [6].

#### 3.9.3 Integrated coping strategies

These strategies combine problem-focused and emotion-focused coping to manage significant ICT events, including SNS usage. Adaptation strategies such as modifying ICT features, usage routines, emotional responses, temporary ICT disengagement, and online/offline venting effectively reduce technostress. Additionally, digital detox has been shown to significantly alleviate stress, irrespective of SNS use intensity [36,54].

Individual and environmental factors, including age, gender, personality, major life events, and general ICT use, further influence the effectiveness of these strategies. Adolescents, for instance, may employ coping strategies that range from emotion regulation and knowledge acquisition to behavior and technology adaptation, aligning with social rules and expectations [36]. This suggests the importance of a context- and population-specific examination of coping mechanisms, particularly among adolescents. Adolescents' coping responses include emotion regulation, knowledge acquisition, behavior adaptation, technology adaptation, and adherence to social rules [36].

While these coping strategies are diverse and adaptable, their effectiveness across various cultural and demographic contexts remains a subject for critical analysis. For example, reliance on temporary avoidance strategies such as digital detox might provide immediate stress relief but does not address the underlying causes of technostress, potentially leading to recurrent stress cycles upon re-engagement with SNS. Moreover, the long-term consequences of persistent SNS use, despite experiencing technostress, include potential escalation into chronic stress conditions. Exploring the long-term effectiveness of these coping strategies and developing comprehensive interventions that consider cultural, demographic, and individual variations in technostress experiences are crucial. Collaboration with SNS platforms to integrate features that promote healthy use patterns and reduce technostress is essential. Such an elaborate approach combines avoidance, active participation, and temporary disconnection to manage personal technostress caused by SNS use effectively. The effectiveness of these solutions depends on individual perspectives, resources, and the cultural and environmental context [2].

## 4 Discussion

This review advances our understanding of SNS-induced technostress by focusing specifically on personal, hedonic uses of SNS—departing from the traditional emphasis on occupational technostress. Unlike prior studies focusing on structured workplace environments with institutional support, this review examines stressors unique to voluntary, personal SNS use, such as social comparison, privacy concerns, and information overload. By addressing this gap, the review fills a critical need to explore the intersection of technological and social stressors in unstructured, non-work settings—a context largely underexplored in existing literature [69].

Building on research by Turel and Qahri-Saremi [67] on SNS fatigue, this review situates technostress within personal, hedonic contexts, where users independently navigate complex stressors. The findings align with the stimulus-organism-response (SOR) model, showing how hedonic motivations for SNS use can lead to technostress and potential disengagement behaviors as users struggle with platform demands [65]. In relation to the SSO (Stressor-Strain-Outcome) model, as emphasized by Maier [1], this review supports the idea that SNS-specific stressors (e.g., social overload and communication complexity) act as distinct triggers for strain in personal contexts. It differs, however, by addressing the absence of workplace coping resources, suggesting that individual coping mechanisms (e.g., digital detox and selective engagement) are critical for mitigating technostress in personal settings. Coping mechanisms, particularly digital detox and selective engagement, are helping users manage technostress in non-work settings. These strategies align with cognitive load theory and dual-process theory. For instance, cognitive load theory emphasizes the importance of reducing mental strain by setting intentional boundaries on SNS use, allowing users to mitigate cognitive burdens associated with continuous notifications and endless scrolling [70]. Similarly, dual-process theory [71] explains the value of selective engagement, which enables users to prioritize deliberate, goal-oriented interactions (System 2) over habitual, automatic checking (System 1), thus alleviating overload and reducing fatigue. This theoretical perspective highlights how coping mechanisms, such as digital detox and controlled engagement, play a critical role in managing SNS-induced technostress when institutional support is lacking.

The interaction of technological, behavioral-technological, and social stressors on SNS further complicates user experiences. For instance, platform complexities, such as frequent interface changes and privacy settings, can intensify social overload, increasing the cognitive effort required to manage online interactions [3,9]. This dynamic creates a feedback loop where anxieties, such as nomophobia (fear of being without one's phone), foster compulsive checking behaviors, heightening stress from privacy concerns and platform invasiveness. The compounded effects of these stressors lead to a range of strains: psychologically, they contribute to anxiety, depression, and impaired cognitive functions [8,52]; behaviorally, they lead to reduced activity or withdrawal from SNS [50]; and physically, they contribute to disrupted sleep, headaches, and eye strain [4]. These findings suggest that SNS technostress has broad implications, potentially deteriorating social capital and support systems across various cultural and demographic groups [58,61].

Theoretical models applied in this review provide a multidimensional framework for understanding SNS technostress. The SSO model helps clarify pathways through which specific SNS stressors lead to strain and coping responses, while the SOR model highlights how hedonic motivations for SNS use drive engagement, thereby exacerbating technostress. Cognitive load theory and dual-process theory further contextualize mental strain from habitual versus intentional use, offering insights into cognitive overload. Self-determination theory (SDT) illustrates how SNS undermines essential psychological needs for autonomy, competence, and relatedness, resulting in emotional strain [72]. Finally, social comparison theory underscores the emotional impact of idealized content on SNS, as users’ comparisons with curated portrayals of others can lower self-esteem and well-being [73].

**Fig. 8:**
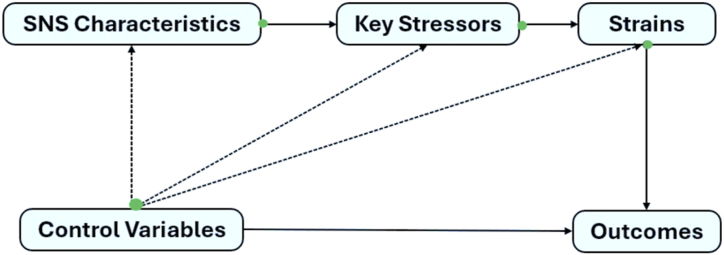
Conceptual model of SNS-induced techno-social stress.

The conceptual model (Fig. 8) illustrates how SNS platforms represent a complex interplay of technological and social stressors, making them a quintessential example of techno-social stress. SNS characteristics (like constant connectivity, social validation, and interface complexities) are not simply technological features but are closely intertwined with social pressures. Another facet of SNS is its rapid evolution, which is closely correlated with how technology improves. Integration of generative AIs such as ChatGPT introduce changes in the way users interact with SNS. As such, there is a constant desire among users to compulsively conform with these changes thereby creating technostress [74,75]. These characteristics introduce key stressors (such as information overload, social comparison, technology anxiety and FoMO), positioning SNS as a central source of stress in the digital age. SNS thus fulfills a dual role as a space for both technological engagement and social interaction, blending these aspects in ways that amplify stress. As users engage with SNS, they experience various strains (psychological, behavioral, and physical) that reflect the pervasive impact of techno-social stress. Psychological strains like technostress and fatigue arise from both the platform's demands and social pressures within interactions. Behavioral strains, such as withdrawal or compulsive checking, further emphasize the unique nature of SNS-induced stress, where users balance between engaging and disengaging for relief.

Control variables such as age, gender, and time spent on SNS modulate these effects, illustrating that techno-social stress impacts individuals differently. For some, frequent SNS use builds resilience or coping skills, while for others, it may heighten feelings of inadequacy or fatigue. These moderating factors shape each user's experience, reinforcing SNS as a nuanced archetype of techno-social stress within the digital landscape.

Ultimately, the culmination of these strains leads to significant outcomes, including reduced well-being, decreased productivity, and in some cases, disengagement from SNS as users seek relief from the persistent stress.

### 4.1 Implications

This review emphasizes the need for tailored strategies to manage SNS-induced technostress across multiple domains to promote mental well-being, productivity, and healthier engagement with digital platforms.

1. Implications for Individual SNS Users

For users, managing SNS-induced technostress is essential for mental health. Technological, behavioral, and social stressors often interact and amplify each other, complicating experiences and creating a cycle where nomophobia and compulsive behaviors heighten stress related to privacy and platform invasiveness [3,9]. This feedback loop can lead to anxiety, cognitive fatigue, and impaired decision-making [8,52].

Users can limit information overload by adjusting notifications, setting SNS schedules, and employing content filters [62]. Practicing mindfulness, setting digital boundaries, and regular "digital detoxes" can help mitigate these stresses [67].

2. Implications for Educators and Students

In education, technostress affects students' focus and academic performance, as stressors like FoMO, social comparison, and constant updates lead to anxiety or depression, impacting learning [8,58,60].

Institutions could implement digital literacy programs covering time management, emotional regulation, and mindfulness techniques. These strategies help students manage technostress, mitigating risks of academic disruption [13].

3. Implications for Employers and Employees

In workplaces, technostress reduces productivity and increases anxiety, especially when privacy concerns and constant connectivity pressure employees to stay accessible. Feature-rich SNS platforms can exacerbate these stressors [3,50].

Employers can adopt policies to moderate SNS use, promote digital wellness, and train employees in mindfulness and self-regulation. For remote workers, guidelines on tech usage and boundary-setting are particularly valuable [21].

4. Implications for Technological Designers

Studies have shown that integration of emerging technologies such as ChatGPT in SNS platforms create digital dependencies, privacy issues and hallucinated content that contribute to technostress [74,76,77]. Designers play a key role in minimizing user technostress through user-centric design. Features like intrusive notifications and complex interfaces exacerbate stress, leading to compulsive behaviors and privacy concerns [4,64].

Designers should incorporate customizable notifications and simplified interfaces. Allowing users to limit notifications and manage engagement can reduce stress and compulsive behaviors, while managing features like "like" counts can decrease FoMO [4].

5 Broader Implications for Social and Cultural Well-being

Technostress has broader social implications, impacting sleep, causing physical symptoms, and leading to social withdrawal. Effects vary across demographics, indicating the need for comprehensive, user-centric solutions [4,58,61].

Technological and regulatory interventions, like intuitive interfaces and digital literacy programs, can reduce technostress. Regulatory policies focused on user well-being could drive systemic improvements for a healthier digital society.

### 4.2 Limitations

This systematic review addresses SNS-induced technostress, synthesizing the variables that cause it, that promote or prevent its emergence, and the outcomes of technostress issues. However, it is limited by the exclusion of grey literature and non-English studies that might have provided additional insights into the global impact of technostress. Also, this review only includes articles from PubMed and Scopus, reputable yet limited in scope. Databases such as Web of Science and publishers’ search engines were not included. This could possibly exclude relevant studies. Future reviews should expand to include additional articles from diverse database sources to capture a broader range of studies on technostress in SNS contexts.

## 5 Conclusion

Technostress is a multifaceted phenomenon that significantly impacts users through SNS, particularly in personal and non-work-related contexts. Despite its pervasive effects, this phenomenon remains under-explored in information systems research. This systematic review addresses this gap by delineating specific stressors, strains, and coping mechanisms associated with SNS use. As integral techno-social systems woven into the fabric of daily life, SNS paradoxically serve as facilitators and stress sources. The omnipresence of SNS, marked by an incessant flow of information and inherent social pressures, often overwhelms users. The stress-relief duality inherent in SNS use underscores a dynamic interplay between enjoyment and fatigue, critically influencing user decisions to continue or disengage from SNS activities. This dynamic was particularly evident during times of heightened societal stress, such as the COVID-19 pandemic, where SNS served both as conduits for excessive information and platforms for emotional support, albeit with potentially deleterious effects on mental health and academic performance.

SNS exemplifies techno-social stress by merging technological features with social interaction, creating unique stressors. Technological stressors pertain to the platform's intricate, unpredictable, and intrusive nature. Concurrently, social stressors arise from the social interactions facilitated by these platforms. These pressures contribute to psychological, physical, and behavioral strains, underlining the intricate relationship between technology usage and its impact on well-being.

Future research should adopt a multi-dimensional approach, exploring how demographic and cultural contexts influence the perception and management of technostress. Specifically, longitudinal studies can track the evolution of technostress over time, revealing its long-term effects and causal relationships. Employing mixed methods would enrich the data pool, integrating qualitative insights with quantitative rigor to provide a more comprehensive understanding of technostress dynamics. An interdisciplinary approach integrating psychology, sociology, and information technology is crucial for developing holistic interventions that effectively address technostress across various life domains. Lastly, as more organizations integrate AI-driven platforms such as ChatGPT in the workplace, a comprehensive review on how these emerging technologies contribute to technostress outside work, specifically in personal and social activities using SNS, will be timely and worth investigating.

## CRediT authorship contribution statement

**January F. Naga:** Writing – review & editing, Writing – original draft, Visualization, Validation, Supervision, Software, Resources, Project administration, Methodology, Investigation, Formal analysis, Data curation, Conceptualization. **Ryan A. Ebardo:** Writing – review & editing, Validation, Supervision, Methodology, Investigation, Formal analysis, Data curation, Conceptualization.

## Data availability

Data will be made available on request.

## Funding information

No funding was provided for this research study.

## Declaration of competing interest

The authors declare that they have no known competing financial interests or personal relationships that could have appeared to influence the work reported in this paper.
